# Microfluidic approaches for the analysis of protein–protein interactions in solution

**DOI:** 10.1007/s12551-020-00679-4

**Published:** 2020-04-08

**Authors:** William E. Arter, Aviad Levin, Georg Krainer, Tuomas P. J. Knowles

**Affiliations:** 1 Department of Chemistry, University of Cambridge, Lensfield Road, Cambridge, CB2 1EW UK; 2 Cavendish Laboratory, University of Cambridge, JJ Thomson Avenue, Cambridge, CB3 0HE UK

**Keywords:** Microfluidic, Approaches, Protein–protein interactions, Diffusional sizing, Electrophoresis, Droplet

## Abstract

Exploration and characterisation of the human proteome is a key objective enabling a heightened understanding of biological function, malfunction and pharmaceutical design. Since proteins typically exhibit their behaviour by binding to other proteins, the challenge of probing protein-protein interactions has been the focus of new and improved experimental approaches. Here, we review recently developed microfluidic techniques for the study and quantification of protein–protein interactions. We focus on methodologies that utilise the inherent strength of microfluidics for the control of mass transport on the micron scale, to facilitate surface and membrane-free interrogation and quantification of interacting proteins. Thus, the microfluidic tools described here provide the capability to yield insights on protein–protein interactions under physiological conditions. We first discuss the defining principles of microfluidics, and methods for the analysis of protein–protein interactions that utilise the diffusion-controlled mixing characteristic of fluids at the microscale. We then describe techniques that employ electrophoretic forces to manipulate and fractionate interacting protein systems for their biophysical characterisation, before discussing strategies that use microdroplet compartmentalisation for the analysis of protein interactions. We conclude by highlighting future directions for the field, such as the integration of microfluidic experiments into high-throughput workflows for the investigation of protein interaction networks.

## Introduction

Proteins form the molecular machinery of life and their interactions control virtually all processes in living organisms (Bork et al. 2004). Between 40,000 and 200,000 protein–protein interactions (PPIs) are believed to exist in the human interactome (Garner and Janda 2011), which are formed by physical contacts between two or more protein molecules. PPIs in both intra- and extra-cellular space are crucial mechanistic features that determine cellular function through DNA replication, transcription, translation and protein folding, as well as activating signalling cascades, controlling enzyme kinetics and facilitating molecular transport. They are responsible for the regulation of the immune system and essential for the vast number of processes associated with cell-surface interactions and extracellular signalling pathways. Conversely, aberrant protein–protein interactions underlie the pathologies of protein misfolding conditions, such as Alzheimer’s and Parkinson’s diseases (Dobson 2003; Chiti and Dobson 2017).

Due to their central role in controlling biological function, PPIs are an area of intense interest for the development of pharmaceuticals with which to modulate cellular processes (Bakail and Ochsenbein 2016). Furthermore, efforts to map the protein interactome, the network of PPIs present within cells, aim to provide an improved knowledge of the complex and subtle variety of PPIs for a deeper understanding of the molecular origins of disease (Ngounou Wetie et al. 2013; Ngounou Wetie et al. 2014). To facilitate these approaches, it is important for PPIs to be identified and characterised. Parallelised microarray (Sutandy et al. 2013), yeast two-hybrid (Lin and Lai 2017) and affinity-coupled MS (Morris et al. 2014) methodologies can be used to identify PPIs in a high-throughput manner: however, these methods are ineffective when quantifying the magnitude of PPIs. This is an important parameter, since the strength of PPIs is related to their biological role. Thus, many techniques have been developed for the quantification of PPIs in terms of the pairwise dissociation constant (*K*_d_) for each interaction. Such methods include classical techniques such as nuclear magnetic resonance spectroscopy (Liu et al. 2016), isothermal titration calorimetry (Krainer et al. 2012; Velazquez-Campoy et al. 2015), gel electrophoresis (Vergnon and Chu 1999; Wittig and Schägger 2009) and surface-based approaches such as enzyme-linked immunosorbent assay (ELISA) (Wittig and Schägger 2009) and surface-plasmon resonance (SPR). Commonly, such methods operate slowly, making them unsuitable for the study of weak, transient interactions in a high-throughput manner and require large quantities of reagents; surface-immobilisation of PPI binding partners can modify their affinity due to inhibition of binding sites (Goebel-Stengel et al. 2011). Furthermore, surface-based approaches are often challenged by non-specific binding and the need for suitable antibody reagents (Güven et al. 2014).

These drawbacks present a demand for experimental techniques that directly assess PPIs in free solution, operate rapidly within a short assay timescale and require minimal sample consumption. In this review, we discuss recent advances in microfluidic methodologies that present potential solutions to these challenges, by enabling rapid, native-state analysis of PPIs. These approaches are often amenable to label-free assay readout, and can be used to improve conventional resonance energy transfer (FRET) or fluorescence-correlation spectroscopy (FCS) experiments (Schuler and Hofmann 2013; Krainer et al. 2019). Microfluidics is a rapidly expanding field based upon the reduction of biological assays to the microscale, in order to access the laminar flow conditions that typify fluid behaviour at these lengthscales (Duncombe et al. 2015; Liu and Liu 2016). Moreover, reagent consumption and experimental timescales are much reduced in microfluidic systems, whilst the potential for assay throughput and parallelisation is enhanced, as described previously (Zhang et al. 2016a, b; Convery and Gadegaard 2019). We thus focus on methodologies that utilise the highly predictable nature of molecular transport under laminar conditions as an analytical tool in of itself, within the context of PPI quantification.

## Essential principles of microfluidics

At the microscale, fluid behaviour differs greatly to that observed in bulk solution (Beebe et al. 2002; Squires and Quake 2005). The low ratio of inertial relative to viscous force at small lengthscales results in laminar flow, where fluid mixing occurs purely through diffusion with complete suppression of chaotic turbulence, which is the primary contribution to mixing in macroscale systems. The laminar regime is characterised by the Reynolds number $\operatorname{Re}=\frac{\mathrm{inertial}\ \mathrm{force}}{\mathrm{viscous}\ \mathrm{force}}=\frac{v\rho L}{\eta }$  (where *ρ* and *η* are the density and dynamic viscosity of the medium, respectively, *v* is the velocity of the fluid and *L* is the characteristic length scale of the fluid movement given by the hydraulic diameter (*d*_*H*_) of the channel. Laminar conditions exist where Re ≤ 1800, ensuring predictable flow conditions for microfluidic applications, where values of *Re*  <<  1 are typical.

The Peclét number $\mathrm{Pe}=\frac{Lv}{D}$ (describes the relative rates of molecular convection relative to diffusion. Typically, microfluidic experiments retain large values of Pe to prevent complete diffusional mixing over the assay timescale. This facilitates experimental strategies that are not feasible in the bulk phase, and means that microfluidic assays intrinsically operate on fast timescales. In bulk experiments, surfaces and solid matrices are required to retain segregation of assay components, whereas under microfluidic conditions, the slow rate of mixing through diffusion alone means that the use of surfaces is not necessary. Furthermore, the physical dimensions of microfluidic devices and the micron-scale nature of molecular transport allow a broad range of experimental lengthscales ranging from Angstroms, as with the study of small molecules, to micrometres in the investigation and manipulation of cellular analytes. Microfluidic techniques are therefore well suited to the study of PPIs in conditions close to the native state. Typically, this is achieved through quantification or manipulation of changes in the size or charge of proteins and protein complexes as they participate in PPIs, by exploiting the diffusion-controlled mass transport of analytes to facilitate analysis of PPI systems as they undergo rapid, in situ changes in solution conditions, or by micron-scale compartmentalisation of assays for high-throughput study of PPI in small volumes, experimental strategies that are the subject of this review. Due to their modular nature, microfluidic devices can be combined for multi-step processes (Mazutis et al. 2009) or integrated with electronic components (Cheng and Wu 2012) and external hardware for mass-spectrometry (Pedde et al. 2017) or synchrotron-enabled spectroscopy (Bortolini et al. 2019), for example.

## Exploiting diffusive mass transport for analysis of PPIs

### Diffusion analysis

As mixing under laminar conditions occurs solely through diffusion (see above), the mixing rate of analytes under microfluidic flow can be analysed to extract the diffusion coefficient $D=\frac{k_BT}{6\pi \eta {R}_H}$ and thus the hydrodynamic radius (*R*_*H*_) of biomolecules. This property has been utilised in the development of techniques for the microfluidic diffusional sizing (MDS) of biomolecules and PPIs. By recording the change in apparent *R*_*H*_ that occurs through protein–protein binding, the presence and strength of PPIs can be observed and calculated. A variety of microfluidic device designs, including T (Kamholz et al. 1999) and H-junction geometries, flow-focussing mixers and capillary-based assay formats such as Taylor dispersion analyses (Chamieh et al. 2017) have been devised to achieve this in practice, yet all essentially function by co-flow of the protein sample through the microfluidic chip alongside a flanking buffer solution. Analysis of the time-evolution of the protein diffusion profile, as it mixes into the co-flow buffer at known fluid linear velocity, thus affords the diffusion coefficient and *R*_*H*_.

**Fig. 1:**
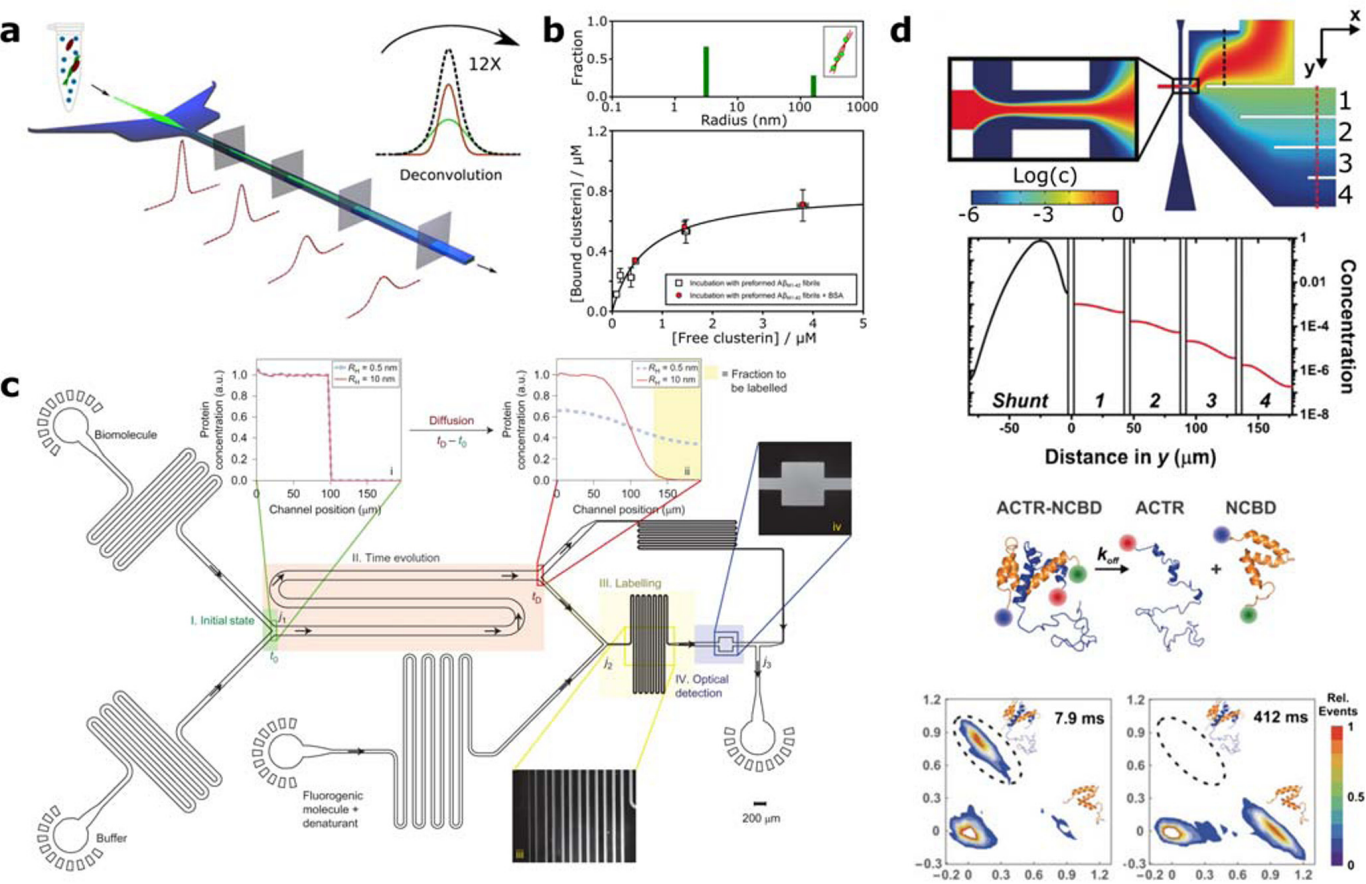
Microfluidic diffusional mixing for the analysis of PPIs. **a** Microfluidic diffusional sizing (MDS) by observation of fluorophore-labelled sample flowing between flanking buffer. The temporal change of the Gaussian fluorescence profile is used to determine the diffusion constant (Arosio et al. 2016). **b** (Upper) MDS data for heterogeneous mixture of clusterin and amyloid-beta fibrils. (Lower) Binding curve for clusterin association to amyloid-beta fibrils generated by MDS (Scheidt et al. 2019). **c** Device schematic for latent-labelling MDS of proteins and PPI systems. Analytes diffuse by an amount inversely proportional to their hydrodynamic radius in the H-filter region (orange), before labelling occur (yellow region) to afford label-free MDS (Yates et al. 2015). **d** (Upper) Device schematic and computed diffusion profiles for rapid sample dilution for smFRET microscopy. Colour scale depicts relative concentration of the analyte. (Upper middle) Analyte concentration for positions shown in the schematic. (Lower middle) Dissociation reaction between proteins NCBD (donor) and ACTR (acceptor) labelled for FRET microscopy. (Lower) FRET histograms for NCBD-ACTR interaction at 7.9 ms and 412 ms after the start of dilution, showing significant complex dissociation within this timescale. Figure taken with permission from Zijlstra et al. 2017

For sufficiently large differences in *R*_*H*_ between PPI binding partners, microfluidic diffusional sizing (MDS) is capable of resolving the sizes and relative concentrations of a range of different protein species (Arosio et al. 2016). This was demonstrated in the observation of the binding interaction between fibrillar alpha-synuclein, an aggregation-prone protein associated with Parkinson’s disease, and a fluorophore-labelled antibody, by flowing the protein sample between two streams of flanking buffer solution in a flow-focussing assay format (Fig. 1(a)). Due to the large difference in *R*_*H*_ between the sample components, the resultant diffusion profile of the protein mixture could be deconvoluted into the separate contributions from both bound and fibril-associated nanobody, thus illustrating the nanobody-fibril PPI (Zhang et al. 2016a, b). Through titration of one binding partner against the other, MDS allows the relative proportion of bound vs. unbound ligand to be determined, an approach employed recently (Scheidt et al. 2019) to quantify the dissociation coefficient between a molecular chaperone and amyloid-beta fibrils (Fig. 1(b)), protein deposits that are implicated in the pathology of Alzheimer’s disease.

MDS quantification of PPIs is particularly facile when binding partners differ greatly in *R*_*H*_, enabling accurate deconvolution of their respective diffusion profiles. However, this feature is not a requirement for accurate analysis of PPIs, which is possible even for binding partners of a similar size, i.e. with values of *R*_*H*_ within one order of magnitude. In this scenario, the average diffusion profile, comprising the diffusive behaviour of all labelled sample components, can be assessed as a function of protein concentration during titration of one element of a PPI pair against the other. This approach was used, for example, for the quantitative investigation of the oligomerisation of molecular chaperone HSP70 (Wright et al. 2018), where real-time observation of aggregation kinetics revealed the co-operative nature of subunit association. A similar method has been employed for immunoassay of an antigen-antibody interaction in whole blood (Hatch et al. 2001). Furthermore, diffusion-based techniques have been employed for the analysis of protein aggregation in antibody preparations (Hawe et al. 2011) and in the development of a wash-free ELISA immunoassay (Kurmashev et al. 2019). Moreover, the continuous-flow nature of MDS makes it suitable for uninterrupted analysis of PPIs occurring in real time, as demonstrated by continuous observation of insulin aggregation by flow-focussing MDS (Saar et al. 2016).

#### Label-free analysis of PPIs in microfluidic systems

In the examples discussed so far, the diffusion of interacting protein species is observed through fluorophore labelling. However, it is well known that fluorophore conjugation can affect the biochemical properties of tagged proteins. Therefore, a number of recent studies have sought to enable label-free MDS, allowing analysis of wild-type protein interactions in the native state. One such approach employs a latent-labelling strategy (Yates et al. 2015), whereby wild-type proteins and interacting protein mixtures undergo size-dependent diffusion in an H-filter device, before in-situ labelling enables quantification of the extent of diffusion and thus the diffusion coefficient (Fig. 1(c)). This method has been used for the observation of a PPI between monomeric alpha-synuclein and a nanobody (Yates et al. 2015), in the study of protein-folding equilibria (Zhang et al. 2018) and in quantifying the phospholipid-dependent binding interaction between peptides relevant to the function of ion channel TRPA1 (Macikova et al. 2019).

An alternative approach to label-free analysis of PPIs is the use of intrinsic fluorescence for the observation of molecular mass transport. By illuminating samples with a UV source at wavelengths below 300 nm, fluorescence imaging of unlabelled proteins is possible through autofluorescence of tyrosine, tryptophan and phenylalanine amino acid residues (Lakowicz 2006). This method has been applied to study the self-assembly of oligomeric alpha-B crystallin (Challa et al. 2018) and protein aggregation of lysozyme and silk fibroin (Toprakcioglu et al. 2019). An additional example of label-free PPI analysis utilised an electrochemical readout, to report a weak binding interaction between free and membrane-bound protein in a microfluidic diffusion-analysis assay (Tan et al. 2012).

### Rapid sample preparation for single-molecule spectroscopy by diffusive mixing

In addition to methods that quantify the diffusion of protein complexes for PPI analysis, the well-defined nature of diffusional mixing in microfluidics allows rapid sample preparation and the analysis of weak, transient PPIs. Through diffusion-controlled mixing, analyte concentration can be reduced over many orders of magnitude within milliseconds, in a predictable and controlled manner. This principle has been used in combination with single-molecule microscopy to probe weakly interacting, intrinsically disordered proteins involved in transcriptional regulation (Zijlstra et al. 2017). Complex formation was conducted at 1 μM concentration, before diffusional mixing rapidly diluted the sample to concentrations below 100 pM within 3 ms. Through single-molecule FRET (smFRET) microscopy, the fast dissociation of PPIs could be monitored, allowing quantitative analysis of the dissociation kinetics (Fig. 1(d)). Importantly, diffusion-controlled mixing enables predictable, rapid sample dilution over μs to s timescales, providing high temporal resolution for the quantification of PPI dissociation in weakly bound complexes. Several studies have used this approach for the investigation of weakly bound PPIs within transient (Gambin et al. 2011) and pre-formed oligomers of alpha-synuclein (Horrocks et al. 2012, 2013; Iljina et al. 2016), for example. Conversely, a recent iteration of the microfluidic-dilution technique utilised a passive mixing unit (Lee et al. 2016) for rapid, diffusion-independent mixing, with complete sample dilution within 20 ms. This allowed temporal smFRET analysis of Hsp90 oligomer dissociation under conditions of constant protein concentration (Hellenkamp et al. 2018).

## Analysis of PPIs by microchip electrophoresis

In addition to passive analysis of PPIs through diffusional sizing, molecular transport can be induced directly by the application of an electric field. Electrophoretic methods have long been a staple technique within molecular biology, which typically make use of a gel matrix in order to prevent the chaotic mixing of sample compounds in bulk-scale experiments. Such techniques usually require a significant quantity of sample, and experiments take minutes to hours to run; biomolecular interactions that do not persist over these timescales are therefore challenging to study. In addition, proteins may interact with the matrix and travel through the gel at different rates depending on the solution composition. Gel electrophoresis relies on measurements of sample migration relative to a set of reference molecules, and results are therefore not readily comparable between solution conditions. Moreover, resolution is insufficient to distinguish between species of similar MW.

To address these challenges, micro-scale electrophoresis techniques have become ever more prevalent. A principal advantage of microfluidic electrophoresis over traditional gel-based methods is that the slow, diffusion-limited mixing present at the micro-scale precludes the need for a gel separation matrix. Thus, biomolecules and biomolecular mixtures can be analysed and fractionated in their native state, with electrophoresis occurring without mediation by a solid support. Due to the micron-scale dimensions and the requirement to maintain high Pe, as outlined above, experiments can be conducted on sub-second timescales, allowing weak and transient biomolecular interactions to be analysed. Furthermore, the lack of matrix means that the electrophoretic mobility $\mu =\frac{\nu }{E}=\frac{qD}{k_BT}=\frac{q}{6\pi \eta {R}_H}$, where *v* and *q* are the electrophoretic drift velocity and net molecular charge, can be determined quantitatively and directly, without recourse to a reference measurement. Moreover, for species of known size, an accurate measure of effective charge can be extracted.

**Fig. 2:**
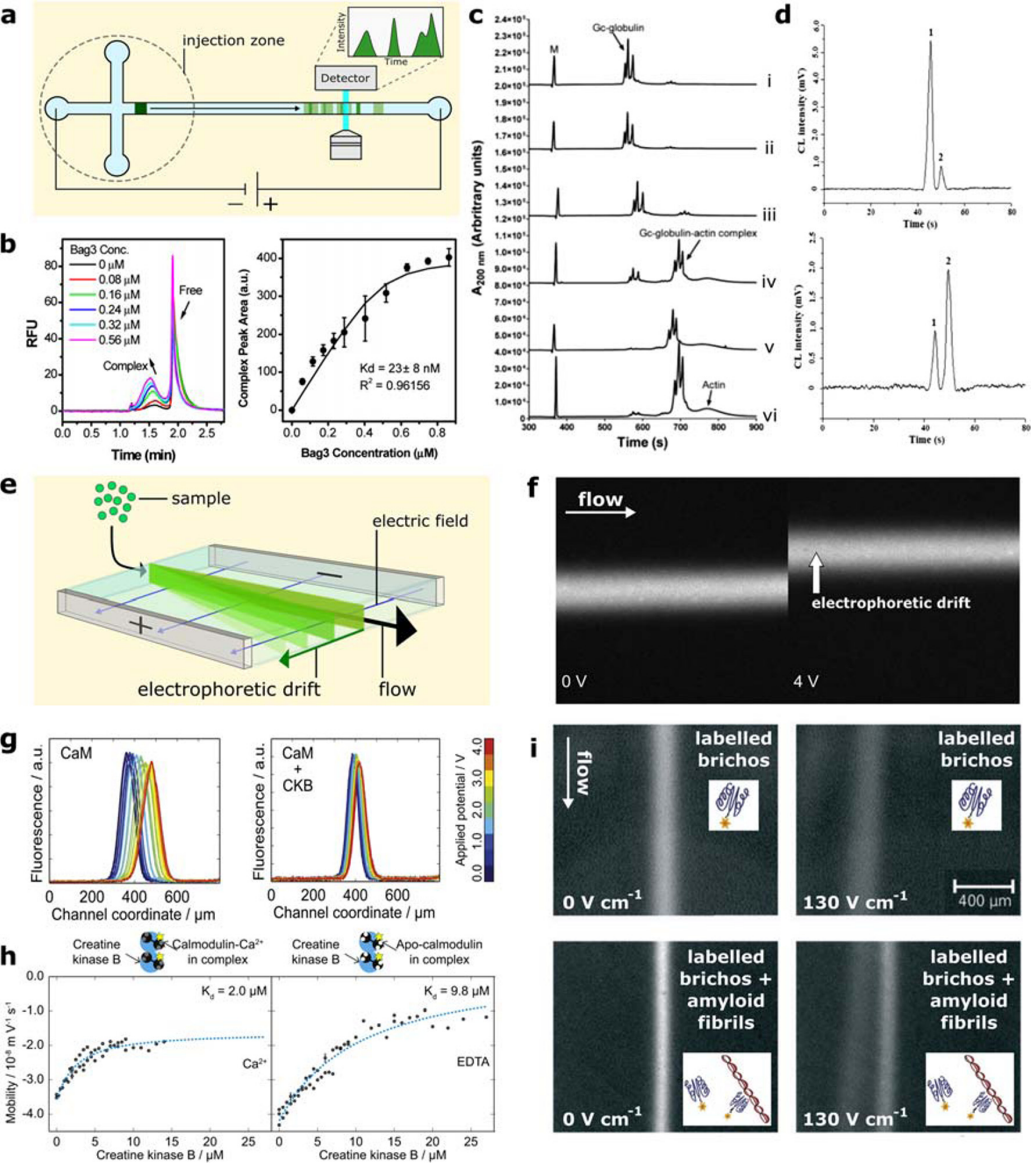
Electrophoretic methods to probe PPIs. **a** Schematic showing operational principle of micro capillary electrophoresis (MCE). **b** Capillary electrophoresis data showing association of BAG3 to HSP70, and corresponding determination of BAG3-HSP70 dissociation constant. Figure taken with permission from (Rauch et al. 2013). **c** Capillary electrophoresis data showing association between Gc-globulin and G-actin, with globulin-actin molar ratios of (i) 1:0.17, (ii) 1:0.22, (iii) 1:0.33, (iv) 1:0.67, (v) 1:1 and (vi) 1:2, respectively. Figure taken with permission from (Pedersen et al. 2008). **d** MCE electropherograms for antibody (1) binding to biomarker alpha-fetoprotein (2) in (upper) normal human serum and (lower) human serum obtained from a cancer patient. Figure taken with permission from (Liu et al. 2017). **e** Schematic showing principle of micro free-flow electrophoresis (μFFE). **f** Micrographs showing electrophoretic deflection of sample stream in μFFE (Herling et al. 2016). **g** Intensity profiles for electrophoretic deflection of fluorophore-labelled calmodulin (CaM) in the absence and presence of creatine kinase-B (CKB) (Herling et al. 2016). **h** Quantitation by μFFE of dissociation constants between CaM and CKB in the presence and absence of Ca^2+^ (Herling et al. 2016). **i** μFFE fractionation of Alexa488-labelled pro-SPC brichos from fibrillar amyloid-beta (Saar et al. 2017). (Upper panels) Labelled brichos only, in the absence and presence of applied electric field perpendicular to flow (left and right panels, respectively). (Lower panels) Labelled brichos in the presence of amyloid fibrils, in the absence and presence of applied electric field perpendicular to flow (left and right panels, respectively)

Micro-capillary electrophoresis (MCE) represents the most commonly applied micro-scale electrophoresis technique, whereby the sample mixture is introduced to a microcapillary before an electric field is applied parallel to the capillary axis, causing electrophoretic migration of the sample components through the microchannel (Fig. 2(a)) (Ouimet et al. 2017; Farcaş et al. 2017; Olabi et al. 2018; Voeten et al. 2018). For example, a capillary electrophoresis platform has been used to quantitatively screen modulation of PPIs by small molecules in a drug-discovery context (Fig. 2(b)). The interaction between chaperone HSP70 and co-chaperone BAG3, in the presence and absence of drug candidates, was observed through MCE fractionation of free and complex-bound HSP70, allowing high-throughput identification of hit compounds (Rauch et al. 2013). MCE has recently been employed for the quantification of PPIs between serum albumins and heparinoids (Mozafari et al. 2018), and for the characterisation of the binding interaction of an anti-lyzosyme antibody and dimerisation of HSP70 through a cross-linking approach (Ouimet et al. 2016). MCE has also been used in the analysis of the PPI between actin-scavenger protein Gc-globulin and free actin (Pedersen et al. 2008) (Fig. 2(c)), and the fractionation and quantitation of aggregated and monomeric amyloid-beta (Picou et al. 2012). Further examples include an MCE immunoassay for the quantitation of cancer biomarker alpha-fetoprotein in human serum (Liu et al. 2017) (Fig. 2(d)), and a competitive immunoassay based upon MCE of anti-thrombin aptamer, for the determination of binding affinities and binding-site identification for distinct anti-thrombin antibodies (Huang et al. 2005).

Furthermore, to MCE, free-flow electrophoresis (μFFE) is an additional subset of microfluidic electrophoresis that has been applied to the investigation of PPIs. μFFE functions by flowing a sample stream, flanked by an auxiliary buffer, through a microfluidic chip whilst an electric field is applied perpendicular to the direction of flow ((Fig. 2(e))) (Fonslow and Bowser 2005: Turgeon and Bowser 2009; Herling et al. 2013; Saar et al. 2017). The resultant electrophoretic deflection of the sample stream can be analysed to afford the electrophoretic mobility, an approach that has been used for the analysis of protein-ion and protein-ligand interactions ((Fig. 2(f, g)). Due to the free-solution, native-state conditions accessible by this method, quantitative values of the absolute electrophoretic mobility and net molecular charge of analytes can be recorded. This method has been used to quantify the binding constant between modulation and creatine kinase and explore the calcium-dependence of this PPI, for example (Herling et al. 2016) ((Fig. 2(h)). Notably, by monitoring the change in mobility caused by protein-association of one binding partner, this approach can determine PPIs even when fractionation of free and complex–bound proteins is not possible, and due to its gentle nature is suitable for the analysis of weakly interacting PPIs. Furthermore, μFFE is capable of analysing interactions such protein–ion binding (Herling et al. 2015) that are inaccessible to size-based methods such as MDS, which are unsuitable in this context due to the negligible increase in hydrodynamic radius that occurs upon association.

As in MCE, sample mixtures may be fractionated during μFFE due to differences in mobility between the sample components (Turgeon et al. 2010; Arter et al. 2018); this approach has been used, for example, to observe binding of pro-SPC brichos, a molecular chaperone, to fibrils of aggregated amyloid-beta (Saar et al. 2017) (Fig. 2(i)). In this experiment, fractionation of the fibril-bound and unbound brichos enables in-situ observation and quantification of the extent of chaperone-fibril binding. As outlined above, such analyses can be carried out with the use of intrinsic fluorescence microscopy, enabling label-free electrophoretic investigation of PPIs (Wright et al. 2019). Due to its ability to continuously fractionate samples, in contrast to sequential fractionation in MCE, μFFE is particularly well-suited to preparative applications followed by down-stream analysis of PPIs (Justesen et al. 2013; Eichacker et al. 2014; Wildgruber et al. 2014). Furthermore, μFFE and MCE has shown promise as a facile method for in-line MS analysis of heterogeneous protein mixtures (Figeys and Aebersold 1998; Song et al. 2010; Park et al. 2015).

## Analysis of PPIs following microdroplet compartmentalisation

**Fig. 3:**
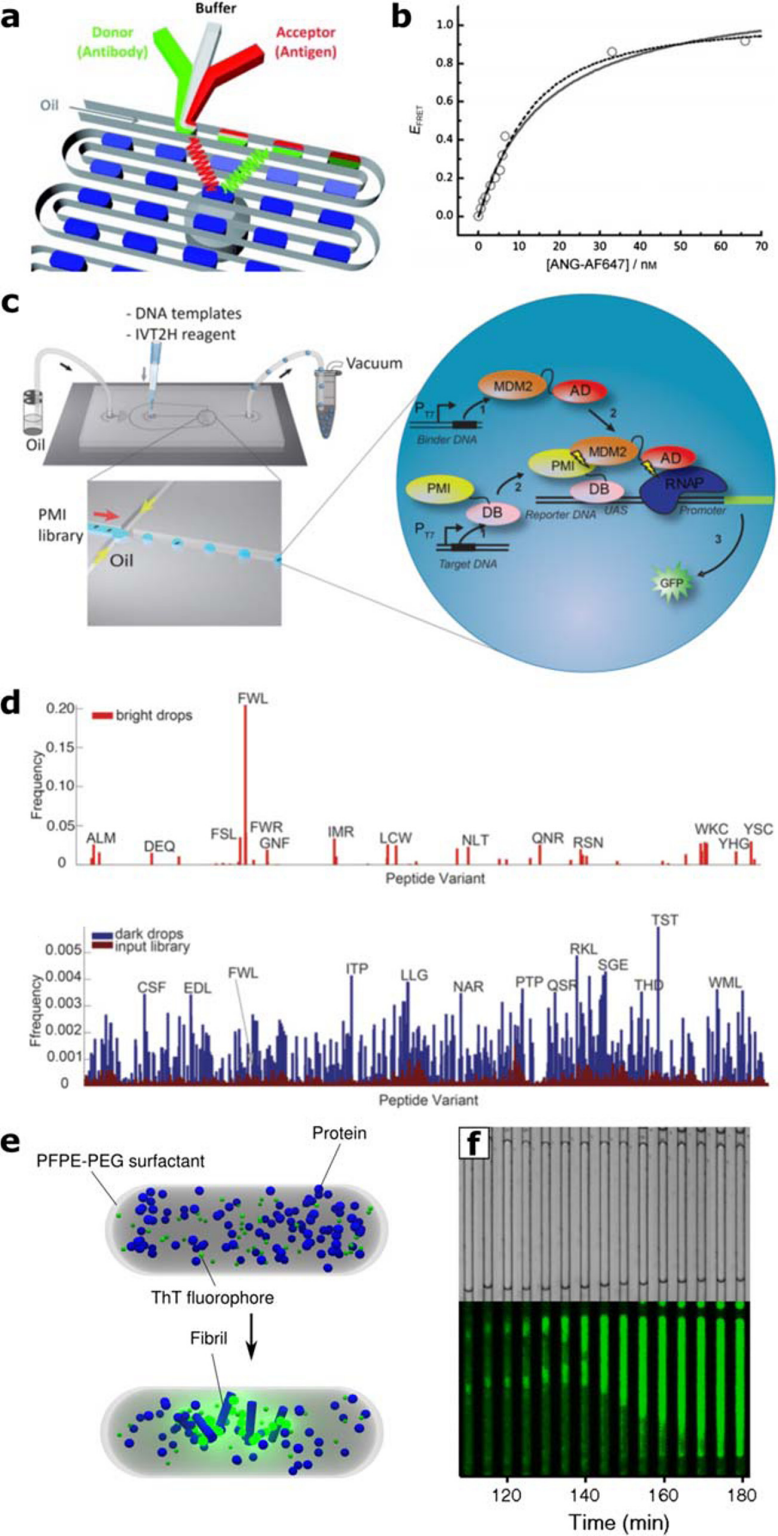
Droplet-microfluidic methods for the investigation of PPI. **a**Droplet-based FRET experiment for the rapid characterisation of angiogenin-antibody binding interaction. **b** Determination of angiogenin–antibody dissociation constant by droplet-FRET. Figures taken with permission from (Srisa-Art et al. 2009). **c** Schematic for single-molecule transcription of short-peptide variants for enrichment of MDM2-binding peptides. **d**, (upper) Identities of high-binding peptides selected by droplet-enrichment relative to (lower) those transcribed from a random library. Figures taken with permission from (Cui et al. 2016). **e** Schematic showing principle for protein aggregation inside micro-droplet environments. **f** Brightfield (upper) and fluorescence (lower) micrographs showing initiation and propagation of insulin fibrillisation in a micro-droplet (Knowles et al. 2011).

In addition to the aqueous-phase microfluidic approaches described above, bi-phasic droplet microfluidics represent another, large class of micro-scale techniques for biochemical, biophysical and biomedical analysis. In micro-droplet experiments, aqueous sample solutions are partitioned into fL to nL droplets by a continuous oil phase and stabilised against coalescence by the presence of surfactant; each droplet represents a unique micro-reactor environment in which biochemical assays can be conducted. Droplets are commonly generated at frequencies of between 0.1 and 1 kHz, although rates of above 1 MHz are feasible (Shim et al. 2013), and droplet compositions can be varied arbitrarily on a drop-to-drop basis (Abate et al. 2010). Thus, droplet microfluidics enable rapid, high-throughput and massively parallel interrogation of biological systems. These properties, together with the fast mixing present in microdroplets (Chen et al. 2018), have been applied to the investigation of a variety of PPI systems. For example, rapid mixing and compartmentalisation, together with FRET microscopy, were used to study the binding kinetics between an antibody and angiogenin (ANG), a polypeptide implicated in angiogenesis and in tumour growth (Srisa-Art et al. 2009) (Fig. 3↓(a, b)). Analogously, rapid quantitation of the ANG-antibody binding interaction and that between chromatin-regulatory proteins and post-translationally modified histone peptides has been achieved via droplet microfluidics, combined with assay readout by fluorescence polarisation spectroscopy [Choi et al. 2012; Cheow et al. 2014).

When present at sufficiently low concentration, assay components can be encapsulated on a single-molecule or single-particle basis (Collins et al. 2015; Mankowska et al. 2016). This enables the high-throughput study of phenomena relevant to PPIs on the single-molecule level, for example in the directed evolution of peptide binders against MDM2, a negative regulator of the tumour suppressor protein p53 (Iwakuma and Lozano 2003; Cui et al. 2016) (Fig. 3(c)). In this study, a library of DNA templates coding for a large variety (i.e. 10^5^ DNA sequences) of short peptide sequences were encapsulated on a single-molecule basis, before candidate peptide species were transcribed in-situ. Using a two-hybrid system (Zhou et al. 2014), the strength of peptide-binding to the co-encapsulated MDM2 target was reported by fluorescence. Droplets displaying high fluorescence were then enriched by droplet sorting (Xi et al. 2017), and their DNA was sequenced to elucidate the identity of the highest affinity peptides for MDM2 binding (Fig. 3(d)).

The small volumes of microdroplets have been further exploited for the study of primary nucleation in protein aggregation. In bulk-phase studies, aggregation reactions are dominated by secondary effects that rapidly amplify the rate of protein misfolding (Knowles et al. 2009), which mask the observation of rare, stochastic primary nucleation events. In one study, the primary nucleation rate of insulin aggregation was quantitatively assessed by confinement of aggregation reactions in pL volumes, so that single nucleation events could be observed on a drop-by-drop basis, an approach that also enabled observation of the spatial and temporal propagation of insulin fibrillisation (Knowles et al. 2011) (Fig. 3(e, f)). Similarly, a study investigated ultrasensitive quantitation of pre-formed insulin aggregates, by employing single-particle encapsulation followed by target-driven signal amplification to observe the presence of amyloid propagons on a digital basis (Pfammatter et al. 2017), with a further work utilising intrinsic fluorescence to investigate protein-misfolding events in microdroplets in a label-free manner (Toprakcioglu et al. 2019).

## Conclusion

The continued investigation of PPIs and interaction networks are crucial tasks for an improved understanding of biological and pathological function. Microfluidic techniques present a facile route towards the analysis of PPIs in a native, label-free manner and on a timescale and in solution conditions relevant to their biological function, whilst requiring minimal sample consumption through the use of low-cost, miniaturised assays.

While a wide range of techniques presented here are in their infancy, even relatively well established methodologies such as MCE are yet to be readily adopted as the go-to technique for standard practice, where techniques such as MS and gel electrophoresis remain the laboratory workhorses. One issue preventing broader application of microfluidic approaches is that many fluidic methods are yet to be integrated into parallelised, high-throughput workflows as have become the norm in e.g. plate-reader assays via robotic automation. Indeed, assay parallelisation represents an essential criteria if microfluidic methods are to find success in the investigation of broader protein interaction networks, rather than being limited to the study of binary PPIs as is presently typical (Liu and Lu 2016). Although microfluidic platforms that enable high-throughput, parallel investigation of PPI networks have been demonstrated (Gerber et al. 2009), these approaches currently require surface-immobilisation of protein reagents. The integration of solution-phase microfluidic techniques, as discussed here, with parallelised, high-throughput assay workflows remains a critical challenge. Furthermore, current efforts to provide meaningful insight into PPIs using microfluidic techniques rely upon strong collaborative efforts in order to bring together the necessary expertise and facilities for both cutting-edge microfluidic studies, involving microfluidic engineering, fabrication and operation and relevant, high-quality experiments in the life sciences. Microfluidic tools themselves may present a means to address challenges regarding throughput and automation, as has been demonstrated by high-speed capillary electrophoresis (Floris et al. 2010; Pan et al. 2018) and the use of droplet microfluidics for rapid front-end sample processing (Ouimet et al. 2019), for example. Moreover, a variety of commercial microfluidic platforms relevant to the study of protein–protein interactions have recently become available, which are predicted to broaden access to microfluidic methodologies to non-expert users in the future (Volpatti and Yetisen 2014; Chiu et al. 2017; Macikova et al. 2019). As this review illustrates, many studies have demonstrated the advantages of microfluidic techniques and their proof-of-concept application to the investigation of PPIs, presenting researchers in the field with the opportunity to adapt microfluidic approaches to their own systems of interest, so as to access parameter space and conduct experiments inaccessible with incumbent techniques.
